# Outcomes of Simultaneous Off-Pump Coronary Artery Bypass Grafting and Vascular Surgery: A Retrospective Case Series

**DOI:** 10.3400/avd.nmt.25-00118

**Published:** 2026-05-22

**Authors:** Keita Kamata, Hideaki Maeda, Masashi Tanaka

**Affiliations:** Department of Cardiovascular Surgery, Nihon University School of Medicine, Tokyo, Japan

**Keywords:** coronary artery bypass grafting, coronary artery disease, peripheral vascular disease

## Abstract

We assessed the outcomes of simultaneous coronary and vascular revascularization in 6 patients with multivessel coronary artery disease and vascular pathologies, including abdominal aortic aneurysm, carotid artery stenosis, and critical limb ischemia. The patients (mean age: 73.6 years) underwent off-pump coronary artery bypass grafting combined with carotid endarterectomy, femoropopliteal bypass, open abdominal aortic grafting, or endovascular therapy. The average surgical duration was 396 min (blood loss: 47–2000 mL), with no major perioperative complications. Simultaneous coronary and vascular surgeries may reduce perioperative complications and eliminate the bleeding hazards associated with dual antiplatelet therapy, while helping to reduce healthcare costs by avoiding staged procedures.

## Introduction

Peripheral vascular disease and coronary artery disease (CAD) commonly coexist, especially in older adults. Up to 50% of patients with significant peripheral vascular disease and carotid artery disease have clinically relevant CAD.^1–3)^ The presence of both conditions complicates surgical planning and increases perioperative risk. Revascularization procedures are traditionally staged; however, this approach may not be feasible for patients with unstable coronary anatomy and critical limb ischemia requiring urgent intervention. We retrospectively evaluated the data of patients who underwent combined off-pump coronary artery bypass grafting (CABG) and vascular surgery at our institution to assess the feasibility, safety, and clinical outcomes of simultaneous surgery for patients with coexisting coronary and peripheral vascular disease.

## New Methods

We retrospectively reviewed the data of all patients who underwent a combined procedure between January 2017 and January 2020.

All patients underwent preoperative coronary angiography and computed tomography of the aorta, lower extremities, and carotid arteries to assess the extent and severity of vascular pathology. The specific vascular condition and number of coronary bypass grafts performed for each patient are provided in **Table 1**. A standardized surgical approach was adopted for all cases. Two dedicated teams (cardiac and vascular) were present for each procedure. The harvesting of the internal thoracic artery and great saphenous vein was initiated under general anesthesia, and the vascular procedures were performed simultaneously.

**Table 1:** Individual patient characteristics

Age/gender	Ejection fractiom	Number of branches	CABG procedure	Vascular procedure
77/M	67.9	3	LITA-LAD, Aorta-SVG-4PD	Above knee-femoral-popliteal bypass
50/M	64.6	3	LITA-LAD, RITA-SVG-4PD	Aorta-bifemoral bypass
84/F	33.9	3	LITA-LAD, Aorta-SVG-HL-PL, Aorta-SVG-#3	Carotid endarterectomy
83/F	85.7	2	LITA-LAD, Ao-SVG-4PD	Carotid endarterectomy
78/F	33.2	3	LITA-LAD, Ao-SVG-4PD, Aorta-SVG-PL	Endovascular therapy
70/M	59.7	3	LITA-LAD, Aorta-SVG-PL	AAA open repair

M: male; F: female; CABG: coronary artery bypass grafting; AAA: abdominal aortic aneurysm; LITA: left internal thoracic artery; LAD: left anterior descending artery; SVG: saphenous vein graft; 4PD: #4 posterior descending branch; Ao: aorta

### Results

Six patients underwent off-pump CABG for myocardial revascularization, followed immediately by abdominal aortic aneurysm (AAA) repair in 1 patient, aorto-bifemoral bypass for arteriosclerosis obliterans in 3, and carotid endarterectomy for carotid artery stenosis in 2. Catheterization was deemed inappropriate for the cases of aorto-bifemoral bypass and above-knee femoro-popliteal bypass in patients with long lesions of chronic limb-threatening ischemia (CLTI), and open surgery was performed. Endovascular procedures were performed via percutaneous catheterization under fluoroscopic guidance. Endovascular aortic repair was deemed inappropriate for AAA open repair owing to a hostile neck, and open surgery was performed. Systemic heparinization was administered to maintain an activated clotting time of >200 s. The selection of grafts and target vessels was individualized based on coronary anatomy. Off-pump CABG was performed in all cases. Anticoagulation was reversed with protamine after all anastomoses were completed. Autologous blood was returned using a cell salvage system in all cases. After confirming adequate hemostasis, both thoracic and vascular incisions were closed, and the patients were transferred to the intensive care unit for postoperative management. For both carotid endarterectomy cases, the carotid procedure was performed first by the vascular surgery team. For all other cases, CABG was performed before the vascular procedure. A detailed summary of each case is provided in **Table 2**. The total duration of surgery ranged from 311 to 453 min (mean: 396 min). The intraoperative blood loss ranged from 47 to 2000 mL (mean: 1061 mL). The average length of hospital stay was 19.5 days. All patients required postoperative rehabilitation and were subsequently transferred to a rehabilitation facility.

**Table 2:** Individual perioperative data

Age/sex	Operation time	Blood loss	Length of hospital stay
77/M	311 min	47 mL	14 days
50/M	424 min	435 mL	47 days
84/F	453 min	1963 mL	20 days
83/F	420 min	306 mL	12 days
78/F	333 min	2000 mL	16 days
70/M	435 min	1615 mL	8 days

M: male; F: female

No major complications occurred in any case during the postoperative period. All patients were discharged on single antiplatelet therapy and were followed up on an outpatient basis. Cardiac function and vascular status were monitored using transthoracic echocardiography and contrast-enhanced computed tomography.

One patient who underwent simultaneous endovascular treatment for superficial femoral artery stenosis experienced stent occlusion at 6 months postoperatively. This resulted in recurrent critical limb ischemia. The patient was readmitted, underwent successful distal bypass surgery, and achieved limb salvage after rehabilitation.

## Discussion

In this study, we employed off-pump CABG for simultaneous surgery. Blood loss was marked and the surgery was prolonged, but blood transfusion requirements were non-inferior, and no postoperative complications were observed. The length of stay for patients who had prolonged hospitalization was not significantly different from that of patients who underwent off-pump CABG at our institution.

CAD occurs in patients with risk factors such as smoking, hypertension, diabetes, and renal dysfunction. The coexistence of CAD is often caused by atherosclerotic changes in the cerebral and peripheral blood vessels. The REACH and AGATHA studies reported that the prevalence of CAR complications in PAD (51.8%) and PAD complications in CAD (21.7%) indicates that both diseases are frequently associated.^1,2)^

Maeda et al. reported that 80% of patients with bilateral carotid artery stenosis of ≥50% have CAD.^3)^ The benefits of simultaneous surgery for each disease have been reported (**Table 3**).

**Table 3:** Summary of previously reported case reports and case series

Authors	Year	Surgical type of vascular surgery	Timing of cardiac intervention	30-day mortality	30-day morbidity
Temizkan et al.^4)^	2014	EVAR to AAA and EVT to SFA (1)	CABG, 5 days after vascular procedure during same hospitalization	0%	0%
Yoda et al.^5)^	2011	Carotid artery endarterectomy (15)	1 Aortic valve replacement and 14 CABGs, simultaneously after vascular procedure	0%	6.7% (1 case: neurological complication)
Wu et al.^6)^	2011	EVTs to the peripheral artery (18)	CABG, simultaneously after EVT	0%	0%

EVAR: endovascular aneurysm repair; AAA: abdominal aortic aneurysm; EVT: endovascular therapy; SFA: superficial femoral artery; CABG: coronary artery bypass grafting

Temizkan et al. performed classical CABG on the fifth day after endovascular therapy for endovascular aortic repair and superficial femoral artery during the same period of hospitalization and reported good outcomes for the treatment of 3 conditions.^4)^ Yoda et al. reported good outcomes for 15 cases of carotid artery surgery with simultaneous valve replacement and CABG using cardiopulmonary bypass.^5)^ Wu et al. also reported that hybrid revascularization was successful in 18 cases without complications.^6)^ Peripheral artery stenting followed by off-pump CABG as a hybrid revascularization procedure is a feasible and less invasive treatment strategy.

Post-percutaneous coronary intervention vascular surgery must be performed with 2 antiplatelet agents for at least a year. Therefore, vascular surgery is not recommended during this period.^7–9)^ However, CLTI can progress within a year, and surgery can be disadvantageous for hemostasis. Simultaneous off-pump CABG and vascular surgery, such as carotid endarterectomy or AAA repair, are feasible for patients with coronary and other vascular diseases. These combined procedures can be performed to simultaneously address multiple vascular issues and streamline the procedure to improve patient outcomes. Careful case selection is essential to balance risks and benefits in patients with complex cardiovascular diseases who have a high perioperative risk.

### Conclusion

Simultaneous vascular surgery and off-pump CABG in patients with severe CAD did not affect the risk of surgical complications and resulted in stable postoperative outcomes. Simultaneous surgeries are useful from both the surgical invasiveness and medical economics perspectives and may represent a useful treatment approach.

## Declarations

### Data availability

The data that support the findings of this study are available from the corresponding author upon reasonable request.
